# Nitrite Dipstick Urinalysis as a Point-Of-Care Test for Sodium Nitrite Poisoning

**DOI:** 10.1097/FTD.0000000000001430

**Published:** 2026-01-09

**Authors:** Corine Bethlehem, Isolde T. Vleut, Dieuwertje Augustijn, Joanne Michielse, Roxane Limmen, Jeroen L. Verweij, Douwe. Dekker, Birgit C. P. Koch, Moska Hassanzai

**Affiliations:** * Department of Hospital Pharmacy, Erasmus MC University Medical Center, Rotterdam, the Netherlands;; † Department of Clinical Chemistry, Erasmus Medical Center, Rotterdam, the Netherlands;; ‡ Center for Lysosomal and Metabolic Diseases, Department of Clinical Genetics, Erasmus Medical Center, Rotterdam, the Netherlands;; § Department of Forensic Investigation, the Police, the Netherlands;; ¶ Public Health Service, Department of Forensic Medicine, GGD Region Utrecht, the Netherlands;; ║ Department of Emergency Medicine—Division of Vital Functions, University Medical Centre Utrecht, Utrecht, the Netherlands; and; ** Dutch Poisons Information Center (DPIC), University Medical Center Utrecht, Utrecht, the Netherlands.

**Keywords:** nitrite urinalysis, sodium nitrite, poisoning, point-of-care test

## Abstract

**Background:**

Intentional sodium nitrite poisoning is an emerging trend worldwide, as it is promoted for its use in self-euthanasia. Nitrite dipstick urinalysis could potentially serve as a rapid-diagnostic tool. Urine dipstick analysis is a common point-of-care test (POCT) for the initial diagnosis of urinary tract infections. The aim of this study was to investigate nitrite dipstick urinalysis as a potential reliable POCT for the early detection of sodium nitrate poisoning.

**Methods:**

Postmortem urine samples, collected over 1 year, were prospectively analyzed for nitrite presence using a urine dipstick method. In addition, a retrospective search was conducted in the database to identify cases with suspected or confirmed sodium nitrite poisoning as the cause of death. Available urine samples from these cases were screened for the presence of nitrite.

**Results:**

Prospectively, 10 of the 420 urine samples tested positive for nitrite presence: 6 samples without any suspicion of sodium nitrite ingestion and 4 with confirmed sodium nitrite poisoning as the cause of death. In addition, in our retrospective study, 12 cases of suspected sodium nitrite poisoning were tested for nitrite presence in the urine using the dipstick. Of these, 8 tested positive for nitrite. Two of the 12 cases were later attributed to intoxication with other substances. As a control, urine samples spiked with sodium nitrite were tested using the dipstick; the pink color intensified with increasing sodium nitrite concentration.

**Conclusions:**

Nitrite dipstick urinalysis is a feasible approach for the early detection of nitrite poisoning in postmortem toxicological screening. In addition, this rapid POCT shows potential as a diagnostic tool for use in acute clinical settings in cases of suspected sodium nitrite poisoning or elevated methemoglobinemia with an unknown cause.

## INTRODUCTION

Sodium nitrite (NaNO_2_) is primarily used as a preservative and coloring agent in food—producing a characteristic pink color—and as an antidote for cyanide poisoning.^1–3^ It is commercially available in most countries. In recent years, intentional poisoning with sodium nitrite has become an emerging trend worldwide.^4–6^ Intentional poisoning with suicide powder (sodium nitrite or sodium azide) has become increasingly common in the Netherlands and surrounding countries as it is promoted for its use in self-euthanasia.^7,8^ There are several case-reports and case-series describing fatal intentional and accidental sodium nitrite poisoning.^9,10^

Sodium nitrite is a powerful oxidizing agent, and ingesting it can result in the formation of methemoglobinemia (MetHb), the oxidized form of hemoglobin (Fe^3+^), which is incapable of binding and transporting oxygen. This can lead to systemic tissue hypoxia with a fatal outcome if left untreated.^1,3^ Furthermore, the conversion of nitrite to nitric oxide (NO) can cause vasodilation and hypotension. Sodium nitrate ingestion can affect vital organs and the clinical symptoms of sodium nitrite intoxication may manifest as cyanosis due to MetHb, hypoxia, altered consciousness, dysrhythmia, and death.^11^ Diagnosing sodium nitrite poisoning in the emergency room typically involves considering environmental factors, assessing clinical symptoms, measuring peripheral oxygen saturation (SpO_2_) levels, and analyzing MetHb blood levels.^4,12^ The treatment of sodium nitrite poisoning consists of hemodynamic support and the administration of intravenous methylene blue, the antidote for increased MetHb in the blood. With the administration of methylene blue, improvement may be observed within minutes.^13,14^ A rapid diagnosis is essential in cases of suspected sodium nitrite poisoning, given its potentially lethal implications and the time-sensitivity of successful treatment. In the Netherlands, a validated method for analyzing sodium nitrate in clinical settings is not currently available. Treatment initiation is based on MetHb levels, and the ability of ambulances and mobile medical teams to assess these levels is limited.

Nitrite dipstick urinalysis could be a potential rapid-diagnostic tool. Urine dipstick analysis is a common point-of-care test (POCT) for the initial diagnosis of urinary tract infections (UTIs) before urine culture results are available. This POCT mechanism is based on the principle that bacteria, if present, can convert nitrates derived from food intake into nitrites in the urine. Consequently, the presence of nitrites in combination with leukocytes is indicative of an UTI or asymptomatic bacteriuria. Substantial amounts of sodium nitrite are excreted by the kidneys.^1,15^ Thus, the detection of nitrite in the urine using nitrite dipstick urinalysis may be a valuable and rapid indicator of sodium nitrite poisoning.

The diagnosis of sodium nitrite poisoning remains challenging in both postmortem investigations and acute clinical settings. As patients with sodium nitrite poisoning are not commonly admitted to the emergency departments of hospitals, due to the high mortality rate, available postmortem urine samples were used in this study. The aim of this study was to investigate nitrite dipstick urinalysis as a potential reliable POCT for the early detection of sodium nitrate poisoning.

## MATERIALS AND METHODS

A prospective study on postmortem material was conducted at the Clinical Pharmacology and Toxicology Laboratory of the hospital pharmacy of Erasmus University Medical Center (Rotterdam, the Netherlands), a tertiary academic hospital. The hospital pharmacy laboratory performs toxicological analyses for approximately >1000 postmortem cases per year. Urine and blood samples from the deceased person are sent to the hospital pharmacy laboratory for postmortem toxicological screening. This is a standard procedure during postmortem external examinations by forensic physicians, as recorded in a local protocol. Analysis is performed within 24 hours of sample collection. The standard toxicological screening of urine includes drugs of abuse test screening (Abbott Diagnostics, Fremont, CA) using the Abbott Architect C4000 analyzer (Abbott Diagnostics). The same analyzer is used to determine the ethanol concentrations in blood samples (plasma) using immunoassay (Abbott Diagnostics). For the toxicological screening of blood (plasma) samples, the blood is analyzed through ultra-performance liquid chromatography–tandem mass spectrometry using the Waters method for toxicological screening, with a library containing more than 1200 compounds. MetHb analysis is performed using a blood gas analyzer (ABL90 series). In this study, postmortem urine samples were prospectively analyzed for nitrite presence using nitrite urinalysis. As the material originated from postmortem cases, no additional approval was required from the institutional ethics committee.

### Prospective Study

For 1 year starting in July 2022, postmortem urine samples were prospectively analyzed for nitrite presence using a urine dipstick method. Urine dipstick (Multistix 10 SG, SiemensMultistix 10 SG, Siemens) analysis for nitrite detection was performed by applying urine to the nitrite reagent pad of the test strip. The color of the test strip was assessed by visual inspection after 60 seconds, as described in the urine dipstick manual. The visual inspection was performed with unaided eyes under normal fluorescent light. A pink color on the reagent pad of the test strip is indicative of the presence of nitrite.

### Retrospective Study

In addition, a retrospective search (2019–2021) of the toxicology laboratory postmortem database was conducted to identify cases with suspected or confirmed sodium nitrite poisoning listed as the cause of death. Available urine samples from these cases were screened for the presence of nitrite using the dipstick method described above.

### Control Samples

Urine dipstick analysis for nitrite detection (as described above) was performed on 3 sets of control samples. Urine samples from patients with (confirmed positive urine culture) and without (confirmed negative culture) an UTI were obtained from the Microbiology and Infectious Diseases Department. The third set of control samples consisted of urine samples from patients with no UTI (confirmed negative urine culture) spiked with sodium nitrite (Ph.Eur.) at concentrations of 0.1–2000 mg/L. All control samples were analyzed for nitrite presence in duplicate.

### Data Collection and Analysis

Data concerning the postmortem persons from whom the urine samples were collected were obtained from the case files provided by the forensic physicians and the toxicology laboratory database. The following variables were collected, if available: age, sex, (suspected) date and time of death, date and time of urine sample collection, conclusion of (un)natural death by the forensic physician, (confirmed or suspected) cause of death, scene findings, manner of death, and toxicology findings including MetHb level. Descriptive data analysis was conducted, and event frequencies were reported. If data were missing or unanswered, they were considered missing values and included in the analysis as “missing.”

## RESULTS

Of 887 postmortem cases screened, the laboratory received urine samples for 553 cases. Nitrite urinalysis was performed on 420 postmortem urine samples (see Table 1 for patient and toxicological characteristics). Among these, 410 urine samples tested negative for nitrite presence. On reviewing the case files, none of these cases involved suspected sodium nitrite ingestion. Ten urine samples tested positive for nitrite presence, evidenced by pink coloring on the nitrite dipstick (see Table 2 for the patient and toxicological characteristics of the 10 nitrite-positive urine samples).

**TABLE 1.** Patient and Toxicological Characteristics of Included Postmortem Urine Samples

	Urine Samples (n = 420)
Age (yr, median)	51
Sex (n)	
Female	79
Male	301
Unknown	40
PMI interval (h, median)	7.5
Natural death (n)	
Yes	32
No	241
Unknown	147
Determined cause of death (n)	
Suicide (unknown manner)	75
Poisoning/overdose	56
Hanging	47
Fall	25
Traffic accident	21
Jump from height	13
Drowning	12
Head injury	9
Suffocation	8
Fall from height	7
Hit by train	5
Gasification	5
Bullet wound	3
Wrist cut	2
Stabbing wound	2
Other	6
Unknown	124

**TABLE 2.** Patient and Toxicological Characteristics of the 10 Prospectively Positive Nitrite Urinalysis Cases

#Case	Sex	Age (yr)	PMI Sampling (h)	MetHb (%)	Presence of Antiemetic in Blood?	Cause of Death	Suspicion of Sodium Nitrite Ingestion?
1	M	67	5	2	No	Hanging	No
2	F	84	11	1	No	Fall from height	No
3	M	44	60	3	No	Suffocation	No
4	M	72	16	1	No	Hanging	No
5	F	61	4	9	No	Fall from height	No
6	M	52	24	1	No	Unknown	No*
7	F	30	7	Unknown	No	Traffic accident	No
8	F	60	7	34	Domperidone	Sodium nitrite poisoning	Yes
9	M	48	3	96	No	Sodium nitrite poisoning	Yes†
10	M	60	72	Unknown	Domperidone	Sodium nitrite poisoning	Yes†

* Case 6 was suspected of alkyl nitrite (poppers) ingestion.

† The ingested substance was provided for analysis. After dissolution, it was tested using the nitrite dipstick, which also yielded a positive result.

On reviewing the case files, in 7 of the 10 cases that tested positive for nitrites (cases 1–7), there was no suspicion of sodium nitrate poisoning, suggesting nitrite presence due to causes other than sodium nitrite poisoning. In addition, 6 of these cases (cases 1–6) exhibited relatively low serum MetHb levels. Notably, case 6 was suspected of ingesting alkyl nitrites (Table 2). For the remaining 3 positive cases (cases 8–10), the forensic physician confirmed sodium nitrite poisoning as the cause of death (Table 2). Two instances of elevated serum MetHb levels were observed in these nitrite-positive cases (cases 8 and 9), further enhancing the suspicion of sodium nitrate poisoning. Toxicological screening of blood samples for cases 8 and 10 showed positive results for the antiemetic domperidone.

Notably, the pink color indicating the presence of nitrite differed in intensity, with the 7 cases without sodium nitrite poisoning displaying a light pink color (cases 1–7), and the cases with confirmed sodium nitrite poisoning displaying a much darker shade of pink (cases 8–10).

### Retrospective Search

**FIGURE 1.**
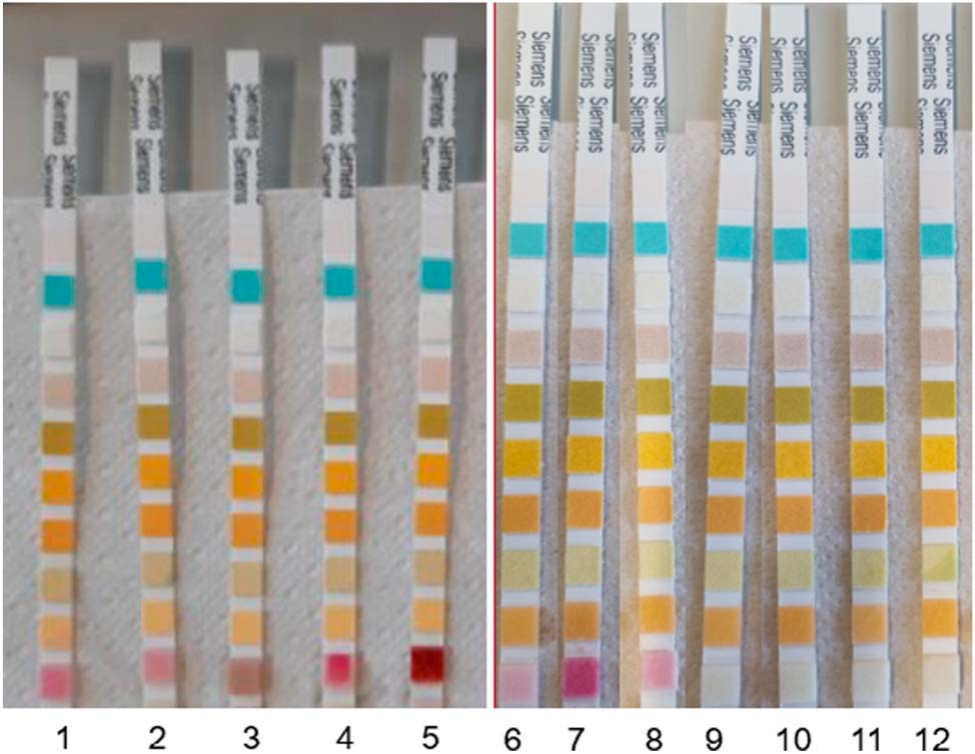
Nitrite urinalysis of urine samples from sodium nitrite poisoning cases identified in the retrospective study, with corresponding case numbers from Table 3.

Retrospectively, 20 suspected cases of sodium nitrite poisoning were identified in the database. Among these, postmortem urine samples were available in 12 cases, which were subsequently tested for the presence of nitrite. Eight of these urine samples (cases 1–8) tested positive for nitrite presence, showing pink coloring (Table 3, Fig. 1), and these cases were confirmed as sodium nitrate poisoning by the forensic physician. Four of these cases also tested positive for an antiemetic medicine.

**TABLE 3.** Patient and Toxicological Characteristics of the Retrospective Study on Suspected Sodium Nitrite Poisoning

#Case	Nitrite Urinalysis Result	Sex	Age (yr)	PMI Sampling (h)	MetHb (%)	Presence of Antiemetic in Blood?
1	Positive	F	48	9	51	Domperidone
2	Positive	M	69	12	49	Domperidone
3	Positive	F	33	98	28	No
4	Positive	F	31	Unknown	94	Metoclopramide
5	Positive	M	19	1	0.0*	No
6	Positive	M	30	3	Unknown	No
7	Positive	M	47	7	Unknown	Domperidone
8	Positive†	M	35	12	Unknown	No
9	Negative	F	81	4	Unknown	No
10	Negative	M	31	14	100	No
11	Negative	M	62	9	6	Metoclopramide
12	Negative	M	58	5	Unknown	No

* MetHb not detectable, patient was treated with methylene blue ante-mortem.

† The ingested substance was provided for analysis. After dissolution, it was tested using the nitrite dipstick, which also yielded a positive result.

Cases 9–12 tested negative for nitrite presence, despite sodium nitrate having been mentioned by the forensic physician as a potential cause of death (Table 3, Fig. 1). Cases 9 and 10 were suspected of sodium nitrite poisoning and the forensic physician noted the time between sodium nitrite ingestion and time of death to be around 30 minutes (with a maximum of 2.5 hours) in both cases. Furthermore, case 10 displayed a 100% serum MetHb level. Cases 11 and 12 were suspected of intentional poisoning, possibly with a suicide powder (either sodium nitrite or sodium azide), by the forensic physician. Case 11 was confirmed to be sodium azide poisoning on laboratory blood analysis for sodium azide, and case 12 was found to involve intoxication with a tricyclic antidepressant.

### Control Samples

**FIGURE 2.**
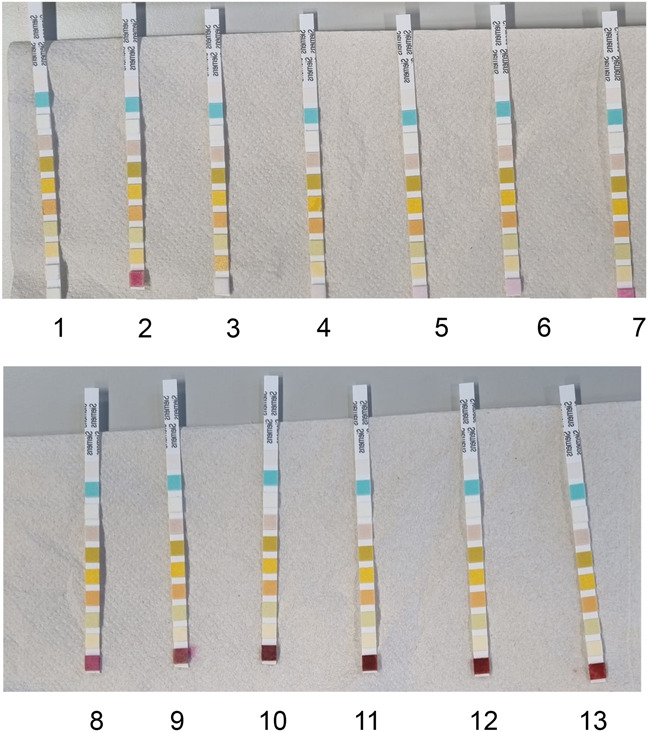
Nitrite urinalysis of control samples. (1) Blanco urine (confirmed negative for urinary tract infection (UTI)); (2) Positive urine (confirmed positive UTI); 3–13: Blanco urine spiked with 0.1, 0.25, 0.5, 1.0, 10, 50, 100, 250, 500, 1000, and 2000 mg/L sodium nitrite, respectively.

The findings from the control samples are depicted in Figure 2. Nitrite was absent in the blanco urine (urine with a confirmed negative UTI). Conversely, nitrite presence was detected in the confirmed UTI-positive urine samples. Increasing the spiked sodium nitrite concentration led to an increase in the intensity of the pink color. Saturation of the pink color occurred at 250 mg/L, with higher sodium nitrite concentrations inducing no further increases in color intensity. The minimum sodium nitrite concentration required to induce pink coloring was 0.25 mg/L.

## DISCUSSION

To the best of our knowledge, this is the first prospective study describing nitrite urinalysis as a POCT for sodium nitrite poisoning in a large cohort. The results demonstrate that nitrite urinalysis has a high predictive value for detecting sodium nitrite poisoning in postmortem urine. Zhang et al^16^ and Stephenson et al^5^ described several case-reports in which nitrite urinalysis was used to retrospectively detect sodium nitrite ingestion. After performing a retrospective case–control analysis of autopsies, Schmitt et al^17^ also suggested the diagnostic potential of the urine dipstick.

Several postmortem processes could influence the formation of nitrites, potentially leading to false-positive results. Increased bacteria postmortem could cause false-positive nitrite results, which may be particularly relevant when the time-lapse between death and specimen collection increases. However, the results of this prospective study do not indicate elevated false-positive results due to an increased bacterial load postmortem, independent of the postmortem interval. In addition, when screening for sodium nitrate poisoning, false-positive results can occur if a patient has an UTI or asymptomatic bacteriuria. In this study, the pink color on the nitrite dipstick reagent pad was more intense in cases of sodium nitrite poisoning than in other cases. The same applied to the tested control samples, with the pink color intensifying with increasing sodium nitrite concentration. Further research is required to confirm whether UTI/asymptomatic bacteriuria and sodium nitrite ingestion can be distinguished based on the intensity of the pink color. Furthermore, compounds containing alkyl nitrites (eg, poppers) can also potentially lead to a positive nitrite dipstick result.^17,18^ We found 1 case of suspected poppers ingestion and observed pink coloring on the dipstick due to the presence of nitrite. In our postmortem toxicological database, there is a lack of alkyl nitrite poisoning cases and cases in which MetHb levels were assessed, warranting future research.

The precise excretion rate of sodium nitrite in urine remains unknown, potentially leading to false-negative results if the urine sample is collected at an early stage of intoxication, or if death occurs shortly after ingestion. However, the short half-life of sodium nitrite (approximately 5 hours) is likely sufficient for a significant amount of nitrite to be excreted in the urine, facilitating the early detection of sodium nitrite ingestion in clinical settings.^19^ In addition, the presence sodium nitrite ingestion symptoms would suggest that the sodium nitrite dosage is substantial enough to result in detectable levels in the urine. In our control samples, a pink color was observable from concentrations of 0.25 mg/L, indicating the high sensitivity of the nitrite dipstick.

False-negatives can also occur owing to the degradation of the nitrite in the urine over time, which could be further enhanced by freeze–thaw cycles. During validation of a laboratory analysis method for measuring sodium nitrite (paper in progress), we noticed a change in the dipstick results over time. Further research is warranted to investigate this effect. Two of our cases from the retrospective search (cases 9 and 10), suspected of sodium nitrite poisoning, were negative for nitrite presence. This could possibly be explained by a short interval between ingestion and death, or by the degradation of the nitrate in the urine samples.

This study has several limitations. First, the urine dipstick test has not been validated for the detection of nitrite following a validated method.^20,21^ As this analysis is not available in the Netherlands, we used serum MetHb concentration as a surrogate marker for sodium nitrite ingestion. In this study, the dipstick was used solely as a screening tool, intended to provide a rapid and accessible means of identifying potential cases that may warrant further diagnostic evaluation. Although it may be valuable for early recognition in clinical practice, it is important to emphasize that the dipstick is not a substitute for more specific and validated diagnostic methods. We are currently developing and validating a quantitative method for sodium nitrate detection. Second, we did not test the negative nitrite urine samples for nitrite presence in the blood or serum MetHb concentrations, potentially resulting in the omission of false-negative results. However, on inspection of the confirmed causes of death in these cases, no (suspected) sodium nitrite poisoning was overlooked. Therefore, missing false-negatives in these cases seems unlikely. Furthermore, we did not test the false-positive nitrite results for UTI confirmation; however, whether UTIs can be reliably confirmed using frozen urine samples remains unknown. Third, MetHb levels in postmortem samples can be falsely elevated owing to autoxidation during storage or falsely decreased owing to MetHb reductase or microbial activity.^22–25^ The latter is linked to the postmortem interval. In our local protocol for postmortem examination by a forensic physician, MetHb analysis is performed within 24 hours (with a maximum of 36 hours) after obtaining the blood samples. Furthermore, we ensured proper storage (direct storage at 2–4°C with preservative) of the collected blood samples, minimizing the influence on MetHb levels. Importantly, the MetHb value alone was not used to determine sodium nitrite poisoning, but considered in the context of the investigation and conclusion presented by the forensic physician and police. Finally, we did not perform the study in antemortem urine samples owing to a lack of these samples. However, we strongly believe that the results of this study can be extrapolated to antemortem settings, as we expect sufficient renal excretion of sodium nitrite in patients showing symptoms of poisoning. Research in clinical patients is required to identify the applicability of this approach in a health care setting.

Notably, the number of positive nitrite cases in this study is not a reflection of the total incidence of sodium nitrite poisoning. The availability of postmortem urine samples is not consistently guaranteed. In our postmortem database, sodium nitrite poisoning is more prevalent than the findings of this study indicate. However, many cases could not be included as urine sampling was not performed when no urine was available. The promotion of self-euthanasia is increasing and most sodium nitrite poisoning cases in this study also involved antiemesis drugs, suggesting that these decedents followed the recommendations regarding self-euthanasia.

This study shows that, as a rapid indicator for the cause of death, nitrite urinalysis can be a valuable tool in postmortem settings. We hypothesize that the added value of nitrite urinalysis is also applicable in clinical antemortem settings. Although therapy initiation should preferably be based on a MetHb value, a MetHb analyzer is not always readily available. Furthermore, for diagnostic purposes, the dipstick can contribute to identifying the cause of elevated MetHb. As demonstrated in this study, nitrite urinalysis is also a useful tool to differentiate between sodium nitrite and sodium azide ingestion in cases of self-euthanasia. Nitrite dipstick strips for urine testing are an easy and accessible diagnostic tool for identifying this highly prevalent and often fatal type of poisoning in post-mortem investigations, and possibly in emergency settings, such as air medical services or ambulances.

## CONCLUSIONS

Urine nitrite dipstick strips provide a valuable supportive tool for the early detection of nitrite poisoning in postmortem toxicological screening. While promising, further validation with a confirmation assay is necessary to ensure reliability. Future research should also explore the applicability of this rapid POCT in acute settings, such as in ambulances, medical helicopters, or emergency departments, when sodium nitrite poisoning is suspected or elevated MetHb of unknown cause is detected.
